# The Utility of ^18^F-FDG PET/CT for Monitoring Response and Predicting Prognosis after Glucocorticoids Therapy for Sarcoidosis

**DOI:** 10.1155/2018/1823710

**Published:** 2018-03-01

**Authors:** Hengyi Chen, Rongbing Jin, Yubo Wang, Li Li, Kunlin Li, Yong He

**Affiliations:** ^1^Department of Respiratory Disease, Daping Hospital, Third Military Medical University, Chongqing 400042, China; ^2^Department of Nuclear Medicine, Daping Hospital, Third Military Medical University, Chongqing 400042, China

## Abstract

Sarcoidosis has significant heterogeneity involving multiple organs; treatment of the disease is a significant therapeutic challenge due to the difficulties in accurately monitoring disease activity and estimating prognosis. Fluorine-18-fluorodeoxyglucose positron emission tomography/computed tomography (^18^F-FDG-PET/CT) plays an important role in assessing the metabolic activity. However, there is not enough evidence about the influence of this method in the clinical management and prognosis prediction for sarcoidosis. This study aims to investigate the clinical utility of ^18^F-FDG PET/CT for therapeutic evaluation and prognostic prediction in sarcoidosis. We had retrospectively enrolled 23 patients with sarcoidosis assigned to receive systemic glucocorticoids. All patients underwent baseline ^18^F-FDG PET/CT before initiating therapy and follow-up ^18^F-FDG PET/CT within 3 months after the therapy. The metabolic and clinical responses were classified. The baseline ^18^F-FDG PET/CT showed increased uptake in all patients. Based solely on biopsy-proven sites, the sensitivity of ^18^F-FDG PET/CT was 91.7%, and the sensitivity improved to 100% after excluding skin involvement. In the subsequent follow-up PET scans within 3 months after glucocorticoids therapy, the SUV_max_ were variously decreased except one; there are significant differences in the clinical remission rates and the relapse rates between patients with a favorable response and cases with no response on follow-up PET scan, the increasing metabolic response was associated with the increase in clinical remission rates and the reduction in recurrence rates. In conclusion, the present study shows that ^18^F-FDG PET/CT is an effective way to monitor the early therapeutic reaction and is helpful in predicting the long-term prognosis of sarcoidosis.

## 1. Introduction

Sarcoidosis is a multisystemic disease characterized by a variable clinical presentation and course, which can lead to severe problems and even death because of advanced pulmonary fibrosis and, less commonly, cardiac, central nervous system (CNS), and hepatic involvement [1]. However, its prognosis is difficult to predict [2]. What is more, monitoring disease activity and response to treatment is difficult in sarcoidosis. Traditional clinical evaluation of patients with sarcoidosis needs many different diagnostic tools, such as chest X-ray (CXR), thoracic high-resolution CT (HRCT), and tests of serum angiotensin-converting enzyme (ACE) concentrations; however, these techniques have been considered to play limited roles [3, 4]. Ga-67 scintigraphy showed multiple additional foci of extrathoracic pathological tracer uptake and pinpointed disease activity, but the low sensitivity of this method also limits its uses [5–7].

Studies have shown that whole-body fluorine-18-fluorodeoxyglucose positron emission tomography (^18^F-FDG-PET) or PET/computed tomography (CT) can be used to accurately assess disease extent and activity and evaluate treatment response [8, 9]; it is more sensitive and useful than Ga-67 scintigraphy in patients with sarcoidosis [6, 10–13]. However, the value of ^18^F-FDG PET/CT in the management and prognostic prediction of sarcoidosis is not known.

Thus, we aimed to investigate the potential usefulness of ^18^F-FDG PET/CT for the response assessment and prognostic prediction of patients with sarcoidosis.

## 2. Materials and Methods

Clinical information was retrospectively obtained from patients with newly diagnosed sarcoidosis in the Daping Hospital between January 2011 and December 2012. The diagnosis of sarcoidosis was based on clinical findings, supported by histological evidence (with the presence of noncaseating epithelioid cell granulomas on biopsies) and after the exclusion of other diseases associated with positive PET findings, such as Wegener syndrome, aspergillosis, and tuberculosis, and malignancies were in accordance with the consensus statement on sarcoidosis of the ATS/ERS/WASOG [1]. Lacking pathological evidence, uncontrolled diabetes mellitus, asymptomatic sarcoidosis, sarcoidosis without glucocorticoids therapy, receipt of other treatment, or lacking ^18^F-FDG PET/CT scan on the baseline and/or within 3 months after systemic corticosteroid treatment were exclusion criteria. Region of interests (ROIs) were selected as those displaying intense uptake on baseline and follow-up ^18^F-FDG PET, and the ROI showing the maximum SUV from these PET slices was taken as SUV_max_. The SUV_max_ were obtained from PET-CT report. Clinical information of patients was obtained by review of the medical record.

The metabolic response was identified as follows: complete metabolic response (CMR): the decrease in SUV_max_ of >75% from baseline; partial metabolic response (PMR): the decrease in SUV_max_ between 25% and 75%; stable metabolic disease (SMD): the decrease in SUV_max_ < 25% or an increase in the SUV_max_ ≤ 25%; progressive metabolic disease (PMD): the increase in SUV_max_ of >25%.

Five roentgenographic stages of intrathoracic changes were recognized according to the classification of Scadding: Stage 0 describes no mediastinal adenopathy or lung infiltrates; Stage I is bilateral hilar lymphadenopathy; Stage II is bilateral hilar lymphadenopathy and pulmonary infiltrations; Stage III is pulmonary infiltrations without bilateral hilar lymphadenopathy; and Stage IV consists of advanced fibrosis with evidence of honeycombing, hilar retraction, bullae, cysts, and emphysema [1]. Clinical remission was defined as the significant improvement of chest radiographs and clinical symptoms (>75% reduction in the subjective perception of intensity of symptoms). Relapse was defined as the progression of clinical symptoms, and/or a radiologic progression.

The Student's *t*-test was applied for comparisons of the measurement data with a homogeneous variance and normal distribution, while the Mann–Whitney *U* test was used for comparisons of the measurement data with a heterogeneous variance and nonnormal distribution. The receiver operating characteristic (ROC) curve was employed to determine the cutoff point for baseline SUV_max_ in order to set apart patients with high risk of relapse. Kaplan-Meier curves were applied to compare the difference of relapse-free survival between groups of different metabolic relapses.

## 3. Results

**Figure 1:**
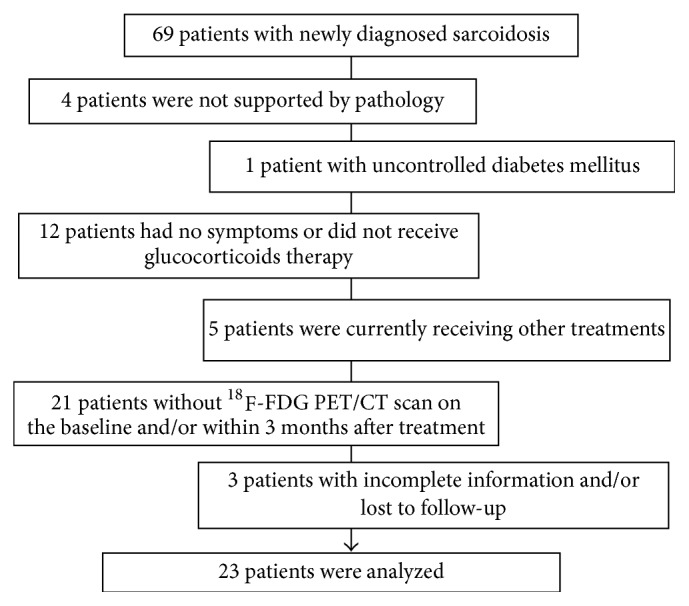
Consort flow diagram.

This retrospective study included 23 patients with clinically diagnosed and histologically proven sarcoidosis (Figure 1); multiple biopsies of skin, thyroid gland, sinonasal mucosa, bronchial mucosa, gastric mucosa, superficial lymph node, and thoracic lymph node were done according to patient's clinical symptomatology and radiological outcomes. The majority of patients had roentgenographic stage I or II intrathoracic change (20 of 23; 87.0%). The demographics and clinical characteristics are shown in Table 1.

^18^F-FDG PET/CT showed increased uptake in all the patients (Table 2). The intrathoracic extent of the PET-positive disease is complete in accordance with roentgenographic stage. Mean (SD) SUV_max_ of positive lesions was 18.6 (7.1) (range, 7.0–31.6). Three patients with CXR stage IV (patients 7, 14, and 20) showed significantly increased uptake in the lung parenchyma. Meantime, ^18^F-FDG PET/CT showed a more extensive disease than HRCT scans in 19 cases; unsuspected extrathoracic sarcoidosis were detected in 7/23, including unsuspected neurosarcoidosis (patient 15). Abdominal and superficial lymph nodes were the most frequent extrathoracic sites found on ^18^F-FDG PET/CT, followed by liver, spleen, bone, and so on. In all 8 patients with biopsy-proven thoracic sarcoidosis, pathological ^18^F-FDG uptakes were detected above lung and/or mediastinal lymph nodes. Moreover, in 15 patients with pathologically proven extrathoracic sarcoidosis (16 biopsy-proven sites of sarcoidosis), ^18^F-FDG PET/CT showed 10 true positives (TPs) in 10 patients with superficial lymph node involvement; 2 TPs in 2 patients with sinonasal mucosa involvement; 1 TP in 1 patient with thyroid gland involvement; 1 TP in 1 patient with gastric mucosa involvement; 2 false negatives (FN) in 2 patients (patients 13 and 18) with erythema nodosum. Twenty-two of 24 biopsy-proven sites of sarcoidosis were accurately detected using ^18^F-FDG PET/CT.

**Figure 2:**
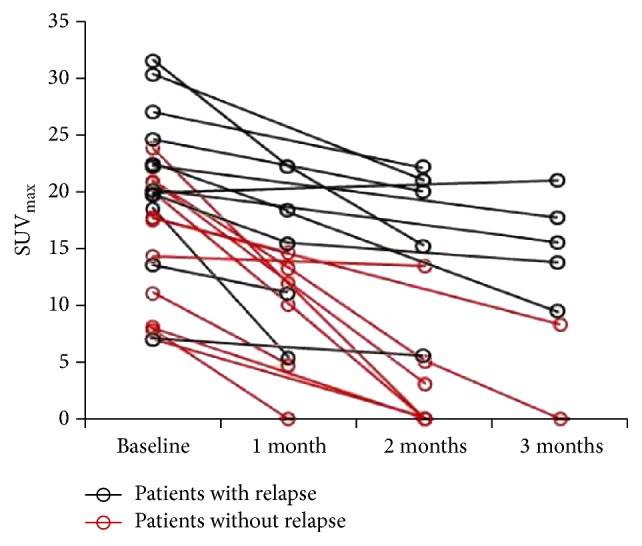
Changes in SUV_max_ for all 23 patients at baseline and follow-up. Compared with that at baseline, the SUV_max_ at 1 month was significantly decreased (*P* = 0.036).

**Figure 3:**
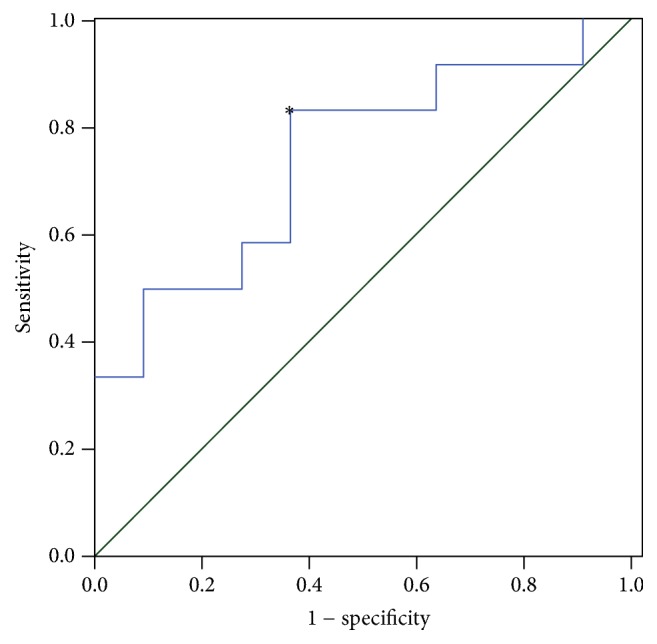
The ROC curve of baseline SUV_max_. The reference line is denoted as the green diagonal. ^*∗*^The optimal cutoff value of baseline SUV_max_.

Based solely on biopsy-proven sites, sensitivity of ^18^F-FDG PET/CT was 91.7%; sensitivity improved to 100% after excluding skin involvement.  Figure 2 shows changes in the representative index for the ROI with SUV_max_ for all 23 patients at baseline and follow-up. The mean baseline SUV_max_ was 15.4  ±  6.0 (23.8–7.0) in 11 patients without relapse, and 21.5  ±  6.8 (31.6–7.1) in 12 patients with relapse (*P* = 0.034). The ROC analysis revealed a SUV_max_ 18.1 as an optimal cut-off level to distinguish between patients with good prognosis and with higher risk of relapse (AUC = 0.742, 95% CI: 0.537–0.948) with a sensitivity of 83.3% and a specificity of 63.6% (Figure 3).

**Figure 4:**
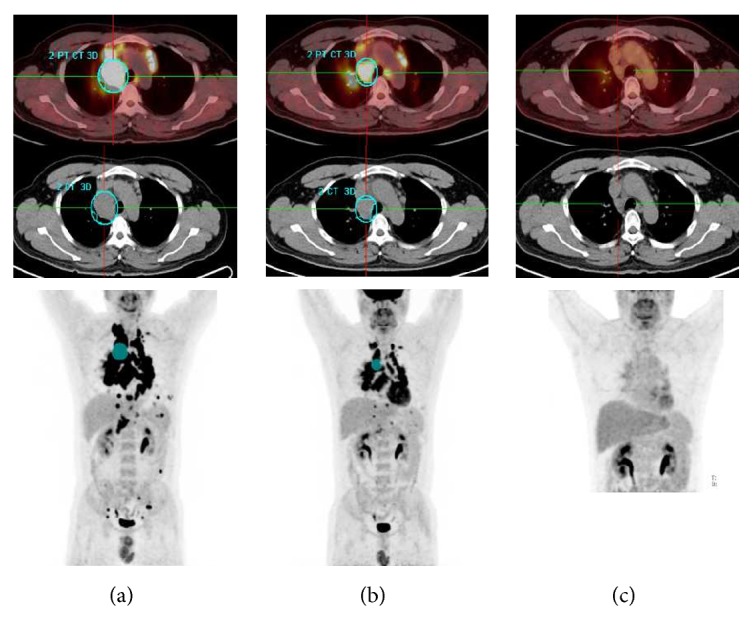
Results for patient 1, a 37-y-old man with symptomatic sarcoidosis. (a) A baseline ^18^F-FDG PET image revealed increased activity in mediastinal lymph nodes, lung parenchyma, liver, and spleen. (b) On the follow-up examination performed at 1 month, PET-CT showed significantly less activity in the mediastinal lymph nodes, liver, and spleen. However, his symptoms persisted but were less severe. (c) On the follow-up examination performed at 2 months, all abnormal uptakes were not apparent.

**Figure 5:**
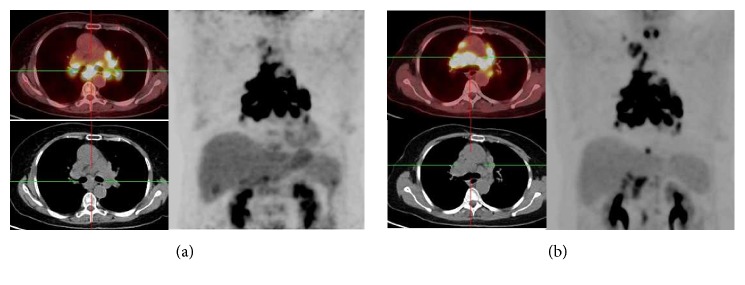
Results for patient 17, a 46-y-old woman with symptomatic sarcoidosis. (a) A baseline 18F-FDG PET image revealed increased activity in mediastinal lymph nodes. (b) On the follow-up examination performed at 3 months, uptake in the mediastinal lymph nodes increased.

In the subsequent follow-up PET scans within 3 months after systemic corticosteroid therapy, the SUV_max_ were variously decreased except patient 17 compared with that at baseline (Table 2); all the lesions with the most intense uptake but patient 7 were the same as baseline PET/CT scan. On the last follow-up PET scan within 3 months, a favorable metabolic response (CMR, 6 patients; PMR, 7 patients) was seen in 56.5% of the patients (as shown in Figure 4), and 10 patients (SMD) had no response on PET scan (as shown in Figure 5).

**Figure 6:**
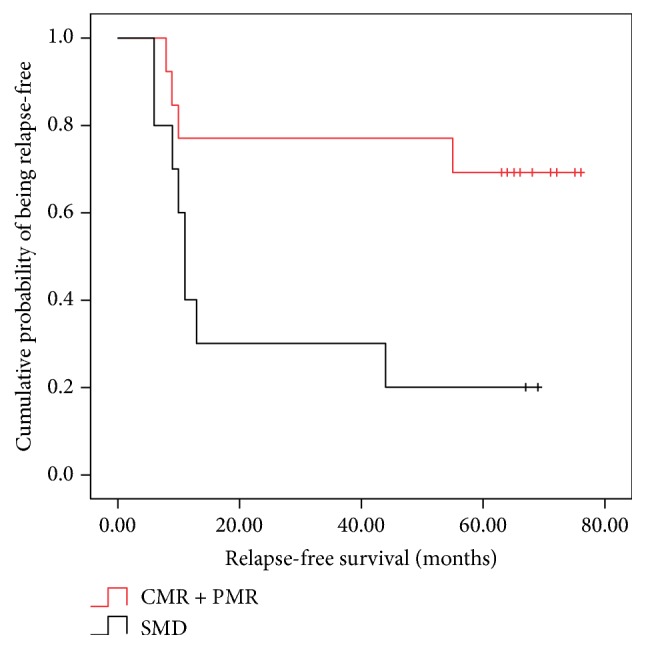
Relapse-free survival analysis. Kaplan-Meier survival curves comparing relapse-free survival of patients with CMR or PMR and with SMD.

Patients with different situations, after taking corticosteroids, had varying degrees of and generally fewer symptoms; some patients could achieve symptomatic improvement. As shown in Table 3, 4 patients with CMR were in clinical remission 3 months after the treatment, and the clinical remission rate rose to 100% within 6 months. With 5-year follow-up, no recurrence was found in patients with CMR. Among 7 patients with PMR, the clinical remission rates were 42.9% and 71.4% within 3 months and 6 months after treatment, which were as low as 10.0% and 40.0% in 10 patients with SMD. Meanwhile, 5-year recurrent rate was 57.1% in PMR patients and it was as high as 80.0% in SMD patients. As illustrated in Figure 6, the Kaplan-Meier survival curves showed a significant difference in relapse-free survival between patients with a favorable metabolic response (CMR and PMR) and those with SMD (log rank test *P* = 0.020) (Figure 5).

## 4. Discussion

Several studies reported a high sensitivity of ^18^F-FDG PET/CT in sarcoidosis [5, 6]. In the current series, ^18^F-FDG PET/CT was contributing the detection of anatomic extent of sarcoidosis, but, more importantly, the baseline SUV_max_ and metabolic response might help to predict the prognosis of sarcoidosis patients with corticosteroids, suggesting that these may be reliable predictors to stratify sarcoidosis patients treated with systemic corticosteroid.

Sarcoidosis may contain numerous different clinical presentations. The rapid diagnosis and the accurate judgment of damage to important extrapulmonary organs and inflammatory activity of pulmonary fibrosis is the fundamental premise of the proper treatment [1, 2]. However, diverse and less typical presentations are present in 50%–75% of cases, which may lead to delayed diagnosis [14]. What is more, for traditional means, noninvasive assessment of the granulomatous inflammatory activity and of the extent of sarcoidosis remains a challenge [15]. Our results showed that ^18^F-FDG PET/CT had a good sensitivity in evaluating disease extent in sarcoidosis and made it possible to study morphological and metabolic changes together. Consequently, it may contribute to initial individualized treatment plans by means of detecting unsuspected crucial extrathoracic diseases especially for asymptomatic patients, and differentiating foci of active inflammatory from lung fibrosis. Additionally, follow-up ^18^F-FDG PET/CT may be particularly useful in diagnosis of sarcoidosis without pathologic evidence for the sensitivity in reflecting the metabolic activity changes, which were associated with the early therapeutic effect of glucocorticoids. Accordingly, the application of ^18^F-FDG PET/CT in the accurate diagnosis and treatment will have a high potential.

Systemic glucocorticoids remain the most commonly used drug for treating sarcoidosis [1]. Timely dose modification (including discontinuation) is conducive to mitigating the side effects, which depend on the accurate treatment response evaluation. However, no clear protocol has been validated for dose and treatment duration [16]. Yamane et al. reported that PET/CT is capable of identifying very early response to treatment in cancer patients [17]. This “therapeutic test” could obviously not be used only in cancer patients; effective treatment could also decrease ^18^F-FDG uptake in inflammation; the quantitative approach using SUV is useful in the estimation of residual inflammatory activity in patients partially responding to anti-inflammatory treatment. In our study, a favorable metabolic response occurred in a part of patients whose clinical remission had not yet appeared. It suggests that ^18^F-FDG PET/CT is a more sensitive parameter of the therapeutic effect than clinical manifestations in sarcoidosis, which may be beneficial in making treatment and regulatory decisions timely and correctly.

Interestingly, it was also found that there are significant differences in the relapse rates between patients with a favorable response and stable disease on follow-up PET scan; the reduction in recurrent rate depended on the increasing metabolic response. The present results indicate that serial assessment of the changes of PET scans is useful in predicting prognosis.

## 5. Conclusion

Altogether, ^18^F-FDG PET/CT is able to detect changes in the magnitude and extent of inflammatory activity during follow-up and plays a unique role in clinical management of sarcoidosis. More importantly, ^18^F-FDG PET/CT may be valuable in predicting long-term clinical outcome of sarcoidosis via the basic SUV_max_ level and the metabolic response to systemic corticosteroid therapy.

## Figures and Tables

**Table 1:** Demographic characteristics of study patients.

	Patients (*n* = 23)
*Age (years)*	50 ± 13
*Male gender, n (%)*	10 (43.5)
*Location of disease, n (%)*	
Thoracic sarcoidosis	3 (13.0)
Extrathoracic sarcoidosis	0 (0.0)
Both	20 (87.0)
*Symptoms, n (%)*	
Fatigue	13 (56.5)
Cough	9 (39.1)
Weight loss	7 (30.4)
Fever	5 (21.7)
Dyspnea	3 (13.0)
Bone pain	3 (13.0)
Chest pain	2 (8.7)

**Table 2:** Patient data, ROIs, and SUV_max_.

Patient	Age (y)	sex	Intrathoracic involvement	Number of ROIs	SUV_max_				Metabolic response	Recurrence time (m)
					Baseline	1 month	2 months	3 months		
(1)	37	M	II	4	23.8 (19.2 ± 6.2)	11.9 (8.6 ± 3.7)	0.0 (0.0 ± 0.0)		CMR	-
(2)	61	F	II	1	11.1	4.8			PMR	-
(3)	62	F	II	2	18.5 (16.5 ± 2.9)	5.4 (4.3 ± 1.6)			PMR	55
(4)	66	F	II	3	30.4 (20.8 ± 11.6)		21.1 (15.7 ± 8.8)		PMR	9
(5)	60	M	I	1	7.8	0			CMR	-
(6)	39	M	I	1	7		0		CMR	-
(7)	46	F	IV	5	31.6 (16.7 ± 11.0)	22.3 (14.6 ± 8.1)	15.3 (9.3 ± 6.4)		PMR	10
(8)	52	F	II	4	24.7 (14.0 ± 10.6)		20.1 (10.3 ± 10.2)		SMD	44
(9)	55	M	I	3	20.0 (10.6 ± 8.3)	10.1 (2.7 ± 4.4)	0.0 (0.0 ± 0.0)		CMR	-
(10)	64	M	I	1	8.1		0		CMR	-
(11)	67	F	I	1	20.9	13.3	5.1	0	PMR	-
(12)	62	F	II	3	22.3 (14.5 ± 6.9)			17.8 (10.2 ± 7.4)	SMD	6
(13)	25	F	I	3	19.8 (13.9 ± 5.2)	15.5 (11.4 ± 3.6)		13.9 (9.7 ± 4.0)	PMR	8
(14)	61	M	IV	1	7.1		5.7		SMD	11
(15)	70	F	I	4	20.8 (10.8 ± 7.1)		3.1 (4.1 ± 4.1)		CMR	-
(16)	47	F	I	7	20.2 (12.3 ± 7.0)			15.6 (9.3 ± 5.5)	SMD	9
(17)	46	F	I	1	19.9			21.1	SMD	10
(18)	47	F	I	3	17.5 (13.4 ± 3.7)			8.3 (5.6 ± 2.4)	PMR	-
(19)	32	M	I	4	27.1 (15.1 ± 8.2)		22.3 (12.8 ± 6.6)		SMD	6
(20)	58	F	IV	2	14.3 (11.1 ± 4.5)		13.4 (11.5 ± 9.6)		SMD	-
(21)	48	M	II	4	22.5 (15.7 ± 9.2)	18.4 (10.1 ± 7.0)		9.6 (5.0 ± 4.3)	SMD	13
(22)	18	M	I	3	17.7 (12.8 ± 4.3)	14.6 (4.5 ± 4.3)			SMD	-
(23)	41	M	I	5	13.6 (9.5 ± 3.2)	11.1 (4.6 ± 3.4)			SMD	11

**Table 3:** Clinical remission and relapse rates of all patients.

	CMR (*n* = 6)	PMR (*n* = 7)	SMD (*n* = 10)
*Clinical remission rates, n (%) *			
3 months	4 (66.7)	3 (42.9)	1 (10.0)
6 months	6 (100.0)	5 (71.4)	4 (40.0)
*Relapse rates, n (%) *			
6 months	0	0 (0.0)	2 (20.0)
12 months	0	3 (42.9)	6 (60.0)
5 years	0	4 (57.1)	8 (80.0)
